# A Survey on Stretching Practices in Women and Men from Various Sports or Physical Activity Programs

**DOI:** 10.3390/ijerph18083928

**Published:** 2021-04-08

**Authors:** Nicolas Babault, Gaelyann Rodot, Marrain Champelovier, Carole Cometti

**Affiliations:** Center for Performance Expertise, CAPS, U1093 INSERM, University of Bourgogne-Franche-Comté, Faculty of Sport Sciences, 3 allée des Stades Universitaires, BP 27877, 21078 Dijon CEDEX, France; gaelrodot@gmail.com (G.R.); marrain.champelovier@gmail.com (M.C.); carole.cometti@u-bourgogne.fr (C.C.)

**Keywords:** training, warm-up, injury, performance, recovery, periodization, methodology, health, wellness, gender, competitive level

## Abstract

Recommendations for prescribing stretching exercises are regularly updated. It appears that coaches progressively follow the published guidelines, but the real stretching practices of athletes are unknown. The present study aimed to investigate stretching practices in individuals from various sports or physical activity programs. A survey was completed online to determine some general aspects of stretching practices. The survey consisted of 32 multiple-choice or open-ended questions to illustrate the general practices of stretching, experiences and reasons for stretching. In total, 3546 questionnaires were analyzed (47.3% women and 52.7% men). Respondents practiced at the national/international level (25.2%), regional level (29.8%), or recreationally (44.9%). Most respondents (89.3%) used stretching for recovery (74.9%) or gains of flexibility (57.2%). Stretching was generally performed after training (72.4%). The respondents also indicated they performed stretching as a pre-exercise routine (for warm-up: 49.9%). Static stretching was primarily used (88.2%) but when applied for warm-up reasons, respondents mostly indicated performing dynamic stretching (86.2%). Only 37.1% of the respondents indicated being supervised. Finally, some gender and practice level differences were noticed. The present survey revealed that the stretching practices were only partly in agreement with recent evidence-based recommendations. The present survey also pointed out the need to improve the supervision of stretching exercises.

## 1. Introduction

Stretching is a very popular and extensively documented [1] exercise modality. It is generally used for health, recreation, and performance. For instance, stretching exercises are implemented in various physical activity programs for therapeutic reasons in different diseases such as rheumatoid arthritis [2] or to correct muscle imbalance in elderly individuals [3]. To date, thousands of scientific papers have been published to determine the acute or chronic effects of stretching [4,5,6,7] and, depending on the objective, to find the best stretching technique [8]. Briefly, stretching exercises are generally used as pre- and/or post-activity routines to increase joint range of motion, health, muscle performance, to promote recovery after exhaustive exercises, or to reduce activity-related injury risks [9,10,11,12,13].

More specifically, numerous studies revealed that flexibility increased with different efficiency after static, dynamic or proprioceptive neuromuscular facilitation (PNF) stretching techniques [14,15,16,17,18,19]. Other derivative techniques such as oscillation could also be used for increasing the range of motion without compromising strength [20]. This well-known increased range of motion and concomitant reduced stiffness are often cited to justify the use of stretching as an injury preventive strategy [10,21]. Muscles are supposed to have an increased ability to resist to excessive elongations [9]. However, evidence for injury prevention remained equivocal and numerous studies presented unclear conclusions [13,22,23,24]. Equivocal conclusions were also obtained when considering recovery. While stretching has been shown to minimize muscle soreness and reduce damaging effects of different metabolites [25], the effects on strength or power restoration remain unclear [12,26,27].

Stretching exercises demonstrated multiple effects on the neuromuscular system. While chronic stretching could, in some situations, enhance strength [28,29], acute effects on strength or power are still debated [11,30,31]. Starting from the late 1990s, authors have concluded that static stretching induced transient decreases in strength [32]. However, numerous stretching parameters have been shown to alter stretching-induced effects on force output. Stretching duration [11,33,34], stretching modality [35], or inclusion inside dynamic activities [36,37] are key factors shown to significantly impact acute strength alterations.

Based on evolving evidence, regularly updated recommendations are made. For example, the Canadian Society for Exercise Physiology recommended using dynamic stretching as a pre-exercise routine instead of static stretching and that static stretching longer than 60 s per individual muscle group should be avoided [10]. Unfortunately, it appeared that coaches or athletic trainers from various sports did not follow these recommendations or recent research findings [38,39]. Nevertheless, this general trend seemed to be reversed. In a recent study, the authors concluded that most soccer coaches from the NCAA adhered to the most recent recommendations [40]. Beside the prescriptions of coaches have been previously explored, to the best of our knowledge, the stretching practices of athletes have not been directly investigated. Athletes’ practices have indirectly been reported by coaches [39]. Because sport or physical activities are not always supervised by any type of sport or health professionals, identifying stretching practices are of paramount importance to give adequate practical guidelines for performance as well as for health benefits. Therefore, the purpose of the present study was to investigate the general stretching practices of individuals from various sports or physical activity programs. We also attempted to determine whether stretching practices were performed empirically or under some professionals’ supervision or at least recommendations. We hypothesized that most individuals have a general practice far from scientific conclusions and that most individuals are not assisted during their practice. Finally, because stretching practices could be influenced by several factors, special attention was given to determine some potential differences depending on the practice level or gender.

## 2. Materials and Methods

### 2.1. Subjects

The questionnaire was electronically distributed, mostly in France, by using different social and personal networks (e.g., universities, sport sciences faculties, sport federations, coaches, physiotherapists, and widely used international social networks). A message was sent with a hyperlink to the online survey. The survey first described key information about the study, its purpose, as well as information related to the research team associated with an e-mail contact. The main criterion for responding the present survey was also defined: being active and regularly practicing sport or physical activities (at least once a week) for competition, recreation, or health. Participants were then clearly informed their written consent was obtained by responding to the survey. The questionnaire and all additional information were in French. The procedure was approved by the local ethical review board (AR2020-08) and was performed in accordance with the Declaration of Helsinki.

### 2.2. Procedure

The online survey consisted of a maximal of 32 multiple-choice or open-ended questions. For some questions, multiple responses were allowed. Depending on answers, the participants had to respond different questions to obtain additional details if necessary. The questionnaire was online for two months (from the very beginning of March to beginning of May 2020). All questions and answers are presented in Supplementary Table S1.

The specifically designed questionnaire was based on extensive discussions, suggestions, and feedback between the research team, coaches, and athletes. Coaches and athletes (~20) were questioned during the questionnaire conception. They were from various sports (individuals or team sports), levels (from beginners to elite athletes), and all obtained high coaching education diploma (for coaches). Also, coaches and athletes from various sports (10) helped the research team to verify the clarity and flow of the survey. They were instructed to respond to the survey according to their real practice and, at the end of the survey, to comment all possible unclear questions or response possibilities. Finally, the content of the final version of the questionnaire was validated by calculating the content validity index (CVI). Eight experts (including coaches and scientists) were requested to rate the relevance of the different items questioned. The content of the present survey was validated with an average scale-CVI greater than 0.91.

The questions of the final version of the present survey covered five main themes: (i) characteristics of the participants such as age, sex, sport, training volume, level, and subjective flexibility evaluation (questions 1–6); (ii) general practices of stretching including the main reasons, the body parts, the frequency and duration (questions 7–16); (iii) stretching education or supervision (questions 17–23); (iv) stretching modalities and their potential effects for performance, recovery, wellness and flexibility (questions 24–29) and (v) injury history and potential effects of stretching on injury prevention (questions 30–32). Additional free-text boxes were used for participants who wanted to provide additional comments. These comments were registered. We arbitrary decided to take into consideration these additional comments when more than 10 participants raised the same comment. Also, care was taken to avoid potential missing responses by using mandatory answers while completing the questionnaire.

### 2.3. Data and Statistical Analysis

Data were first reviewed and incomplete responses or from individuals without any actual sport practice were excluded from analyses. We analyzed the distribution of the response frequencies. Descriptive statistics are reported in the form of percentages and counts. Depending on sex or total sample, percentages were calculated as a function of total respondents for the corresponding question. Subgroup analyses were carried out to compare responses according to sex (women vs. men) and to the practice level (national/international vs. regional vs. recreational). The frequency rates were compared using two-tailed Chi-square tests with the significance level set at *p* < 0.05. The Cramer’s V scores illustrating the effect sizes were also calculated from the chi-square and presented in Supplementary Table S2. In case of significant Chi-square, pairwise comparisons were achieved by calculating Z-scores. Statistics were performed using JASP (Ver 0.13, JASP Team (2020), University of Amsterdam, Amsterdam, The Netherlands) and SPSS (Ver 27, IBM-SPSS Inc., Armonk, NY, USA).

## 3. Results

### 3.1. Characteristics of Participants

A total of 3572 responses were obtained. Twenty-six responses were excluded from the analyses because the respondents did not meet the main inclusion criterion (training at least once a week). A total of 3546 questionnaires were therefore analyzed. Statistical analyses indicated significant different frequency distributions between gender and practice level for age, sport, level, training volume, and subjective flexibility (*p* < 0.001) (Table 1). Briefly, women were younger, mostly recreative with shorter training volume per week (<6 h) and with higher flexibility than men. The frequency was greater for women than men for Dance/Gymnastics, Equestrian/golf, Fitness and Swimming while it was greater for men for Cycling/trail/triathlon, Racket sports, and Team Sports. Finally, recreative individuals mostly performed fitness and strength of long-duration activities.

### 3.2. General Stretching Practice

**Figure 1:**
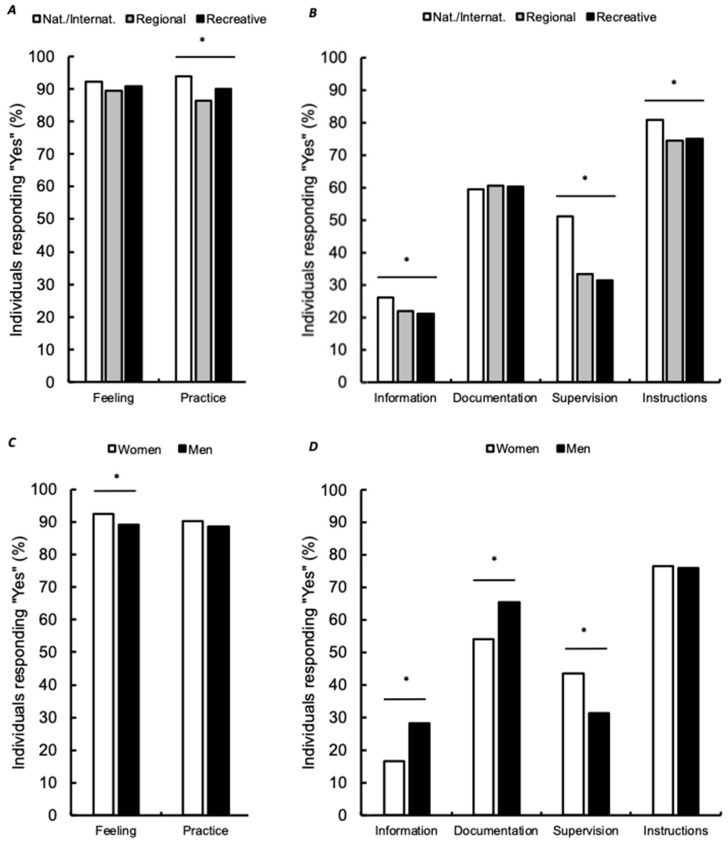
Percentages of “yes” to different questions. Feeling: Do you already feel the necessity to do stretching exercises? Practice: Did you conduct stretching exercises during the last two years? Information: Did you receive any information about stretching during your education? Documentation: Did you look at some documentation to help you understand and perform stretching? Supervision: Are stretching exercises supervised? Instructions: Do you have instruction to perform stretching? Feeling and practice are shown as a function of practice level (**A**) and gender (**C**). Information, documentation, supervision and instructions are shown as a function of practice level (**B**) and gender (**D**). Percentages of “yes” are shown and expressed as a function of the total number of respondents for a given practice level or gender. Significant frequency distribution differences obtained from chi-square and Z-scores between women and men or between nat./internat. and both regional and recreative individuals are shown (* *p* < 0.001). Nat./Internat.: national/international level.

Most respondents indicated they felt the necessity to stretch and conducted stretching during the last two years (Figure 1). Frequency distribution revealed more “yes” responses for women than men (*p* < 0.001) but no difference was observed for the different practice levels (*p* = 0.139). Briefly, individuals mostly indicated it was a necessity because of muscle pain (59.6%), muscle stiffness (59.0%), or simply for wellness (60.0%). The majority indicated it was a necessity after training or competition (77.9%) or after a series of training or competition (32.6%). No difference in distribution was obtained between women and men for these two last questions (*p* = 0.092 and *p* = 0.074, respectively). In contrast, significant frequency distributions differences were obtained depending on the practice level (*p* < 0.001).

Almost similarly, most respondents performed stretching within the last two years (Figure 1). The responses were not different between women and men (*p* = 0.108) but individuals in a regional level performed less stretching than those recreational and performing at a national/international level (*p* < 0.001). Participants who did not conduct stretching indicated it was because of a lack of motivation (26%), time (22%), knowledge (why and how to do, 20% and 13.7%, respectively), lack of supervision (10.3%), or poor efficiency (6.4%). Individuals performing stretching mostly responded it was for recovery, to gain flexibility, for injury prevention and performance (Table 2). As compared to men (*p* < 0.001), women mostly indicated it was for recovery. Comparing practice level revealed, recreational individuals mostly conducted stretching exercises for wellness while competition athletes mostly looked for injury prevention and warm-up effects (*p* < 0.001). Stretching was mostly performed after training, then in dedicated sessions or before training. Women distribution was greater for the answer “after training” than men (*p* = 0.006). Recreative individuals mostly performed stretching after training/competition (*p* < 0.001). Stretching frequency was mostly “1 to 5 times a week” and “during every training”. No difference was obtained between women and men (*p* = 0.121). National/international individuals mostly practiced stretching every day than the others (*p* < 0.001). Stretching sessions were mostly shorter than 15 min without any difference between women and men (*p* = 0.414) but with shorter stretching sessions for competitive athletes as compared to recreational individuals (*p* < 0.001). Finally, stretching was mostly performed over the whole body with significant differences between women and men (*p* < 0.001).

### 3.3. Education and Supervision

Most respondents indicated not receiving any information during their education (Figure 1) but that they often looked for information (~60%) while reading books (45.0%), discussing with others (47.0%), or surfing the internet (34.5%). Two third of the individuals are not supervised during stretching. Women indicated they were more supervised than men (*p* < 0.001). Similarly, national/international levels individuals indicated being more supervised than the other levels (*p* < 0.001). Stretching was mostly supervised by coaches (95.3%) then by health professionals (34.5%) and other athletes (24.7%). Responses were similar between women and men (*p* = 0.134) but coaches mostly supervised stretching sessions in national/international individuals as compared to the other practice levels (*p* < 0.036). In contrast, most individuals responded receiving instructions (76.2%). No difference was obtained between women and men (*p* = 0.641) but national/international individuals obtained much more instructions than the others (*p* = 0.002). Instructions were mostly given by coaches (92.8%) or health professionals (61.7%). Instructions were also given during individuals’ education (25.7%) or by other athletes (16.9%). No difference was obtained between gender and practice level (*p* = 0.086 and *p* = 0.099, respectively).

### 3.4. Stretching Modalities

**Figure 2:**
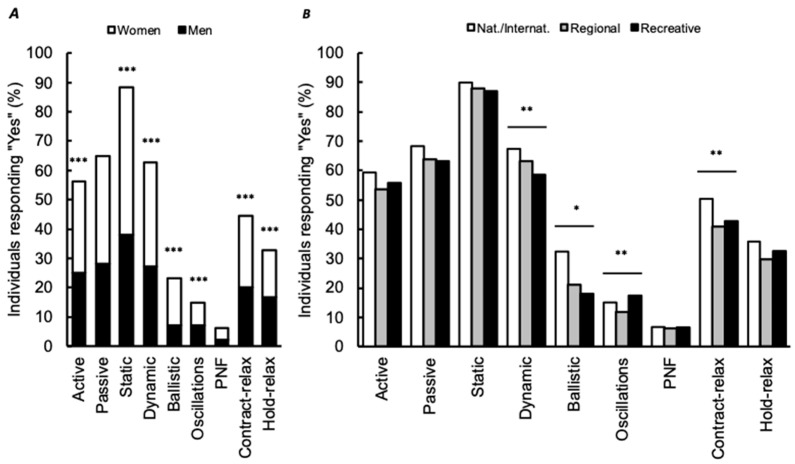
(**A**) Stretching modalities generally used as a function of gender. Percentages of “yes” are shown and expressed as a function of the total number of respondents. Repartition between women (black part of histogram) and men (white part of histogram) are also shown. (**B**) Stretching modalities generally used as a function of practice level. Percentages of “yes” are shown and expressed as a function of the total number of respondents for a given practice level. Significant frequency distribution differences between gender or practice level are shown (*: *p* < 0.05, ** *p* < 0.01 and *** *p* < 0.001). For oscillation, nat./internat. individuals were not different from recreative (Z-score results). For contract-relax, no difference was obtained between regional and recreative individuals (Z-score results). PNF: proprioceptive neuromuscular facilitation. Nat./Internat.: national/international level.

**Figure 3:**
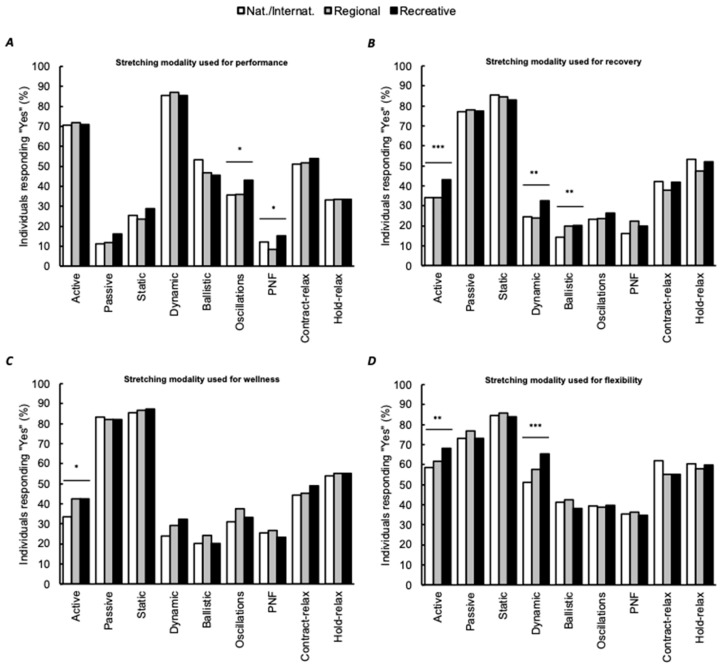
Stretching modalities used for performance enhancement (**A**), for recovery (**B**), for wellness (**C**), and for flexibility (**D**). Percentages of “yes” are shown and expressed as a function of the total number of respondents for a given practice level. Significant frequency distribution differences between all practice level are shown (*: *p* < 0.05, ** *p* < 0.01 and *** *p* < 0.001). For panel **A**, no difference was observed between nat./internat. and regional for oscillation and between nat./internat. and recreative for PNF (Z-score results). For panel **B**, nat./internat. were not different than regional for active and dynamic stretching and no difference was obtained between regional and recreative individuals for ballistic (Z-score results). For panel **C**, no difference was obtained between regional and recreative individuals (Z-score results). PNF: proprioceptive neuromuscular facilitation. Nat./Internat.: national/international level.

In our study, 61.7% of the participants indicated knowing different stretching modalities. The distribution of “yes” was greater in men than women (68.3% and 54.5%, respectively, *p* < 0.001) and greater in national/international than recreative and regional individuals (70.0%, 64.9% and 54.9%, respectively, *p* < 0.001). For individuals responding “yes”, additional questions were provided. Individuals had to indicate whether they generally used these techniques (Figure 2) and to specify the expected effect (performance, recovery, wellness or flexibility; Figure 3). 

### 3.5. Injury

In our study, 45.1% of the respondents reported getting injured during the last 12 months (Table 3). Injury was more present in men than women (*p* < 0.001) and less in recreative individuals (*p* < 0.001). In case of injury, 47.4% responded that performing more stretching would not have help them avoid being injured. National/international individuals mostly indicated stretching would not have avoided their injuries (*p* = 0.004). In case of no injury, 84.9% responded stretching was efficient to avoid injury or was likely efficient to avoid injury. Significant differences were observed between women and men (*p* = 0.003) while no difference was obtained between practice level (*p* = 0.172).

## 4. Discussion

The present study aimed to investigate the stretching general practices of athletes from various sports or physical activity programs. Our results revealed that the large majority of athletes are not supervised during stretching exercises, but most received instructions from their coaches and are looking for information. Most conducted stretches at least once a week with stretching sessions lasting less than 15 min, generally for recovery after training sessions or competitions. Only 61.7% of the respondents knew the existence of different stretching modalities. From these individuals, and contrarily to our hypothesis, most favored the use of dynamic stretching for performance purposes and preferred static stretching for flexibility, recovery or wellness. These general observations are partly concordant with the literature [10,31,40]. Beside these conclusions seemed to reveal that athletes generally followed stretching evidence-based recommendations, one should remember that 38.3% of the respondents were unable to differentiate stretching modalities or terminologies.

### 4.1. Stretching Practices

The respondents of the present survey mostly felt the necessity to stretch to improve flexibility and wellness. Firstly, it is well known that stretching increased flexibility (i.e., range of motion) and/or decreased stiffness [6,41,42,43]. Secondly, the assumptions for health and wellness benefits were generally concordant with the literature. For instance, multiple studies have tested the effects of stretching programs implemented in office settings [44,45,46,47]. These studies were generally conclusive for significantly improved health-related quality of life. However, the time to gain ratio could be questioned. Indeed, stretching is only a part of general fitness programs that should include other components such as strength or endurance. In a very recent paper, the author suggested to retire flexibility from fitness programs [48] so as to partly save time and emphasize the other components that could have more robust benefits for health.

Individuals also responded feeling the necessity to stretch to reduce muscle pain. This result was congruent with the literature since recent evidences demonstrated the positive effects of stretching on pain sensitivity [49] with potential roles in endogenous pain inhibitory systems [50]. For that reason, 74.9% of the respondents indicated using stretching for recovery (i.e., performance or muscle soreness) and therefore after single or multiple training sessions. However, no clear evidence demonstrated the positive effects of stretching for recovery. Some authors observed small-to-moderate effects on perceived muscle soreness and recovery of muscle function after eccentric exercises [51]. In contrast, numerous studies demonstrated stretching was ineffective to decrease muscle soreness [12,52,53] or prevent cramping [54]. When performed during inter-set recovery periods during resistance training, stretching even negatively impacted neuromuscular performance [55]. From the present results, we concluded that athletes apply stretching for recovery while the effects are not clearly evidenced by the scientific literature.

A similar conclusion is obtained while considering injury prevention. More than half of the respondents indicated performing stretching for injury prevention. Surprisingly, the practices and beliefs were somewhat conflicting. Indeed, only 38.4% indicated stretching was efficient for injury prevention (29.7% did not link stretching and injury and the remaining 31.9% did not know). Interestingly, the results of the present survey for injury were coherent with the literature. Indeed, while injury prevention was often cited to justify the use of stretching during pre-activity warm-up routines [5,9,10], the effects were generally unclear with only limited beneficial results [13,56,57,58,59]. In addition, some authors [58] indicated injury incidences were greater in very stiff or very flexible individuals. This suggested that individuals should regularly conduct stretching programs (as revealed here in most individuals) to gain or, at least, to maintain flexibility. This potential link between flexibility and injury prevention was reflected by the present survey. While more women indicated stretching contributed to the absence of injury as compared to men, women and elite individuals indicated that additional stretching would not have avoided injuries. We could speculate such response could be attributed to the subjective flexibility. Indeed, although the direct link could not be verified, women and elite individuals generally estimated their flexibility as higher than the others. In addition, these individuals stretched slightly more frequently while mostly responding looking for gains of flexibility than men or regional level athletes. Obviously, additional analyses should be conducted to link very detailed stretching programs (e.g., frequency, stretching technique, timing) with the type of injury but also with the sport actually practice, training volume or age.

When performed during warm-up (before training or competition), the reasons for using stretching were performance improvement, range of motion gains, or injury prevention. The question of the effectiveness of stretching for performance is currently widely documented. The literature generally agreed with the fact that short durations stretching exercises could be performed within a comprehensive warm-up procedure [5,10,37] and that dynamic stretching (slow conducted dynamic stretch) is recommended [35]. Obviously, for some sports (such as gymnastics), flexibility is part of the performance determinants and stretching should be included as a pre-exercise routine. Hopefully, the authors previously demonstrated that the potential acute detrimental effects of stretching are lower in individuals with greater flexibility [60]. Taken as a whole, these results were contradictory with our initial hypothesis but were concordant with scientific recommendations since individuals mostly reported stretching using short sessions with predominant dynamic or active stretching modalities [61]. Such encouraging finding is consistent with the general coach practices recently documented [40]. Considering older studies [39,62], it suggests that beliefs are positively evolving towards evidence-based practices.

Finally, half of the respondents indicated they performed stretching in dedicated sessions. Interestingly, these specifically designed stretching sessions might favor flexibility improvements while being focused on this training component. Moreover, it could limit some detrimental effects of stretching (such as recovery or acute force decrements). For instance, when stretching is performed for recovery, authors have suggested that it could even have additional negative impacts leading to delayed onset muscle soreness (i.e., eccentric) [63,64]. Also, it might alleviate some detrimental performance effects when long duration stretches are programmed [65]. However, whether stretching should be recommended during dedicated sessions requires further investigations.

### 4.2. Education and Supervision

From the sample considered, and as previously stated, most respondents performed stretching exercises. Very few individuals received information during their education and about half are looking for information while discussing with athletes, coaches or health professionals. However, from the present survey, we cannot estimate the quality of the information received (whether information is up to date). For instance, information could more frequently be related to personal experiences (history of practices or empirical beliefs of potential benefits or drawbacks) rather than scientific evidences [66,67].

Consistent with our hypothesis, the present results indicated that most individuals are not supervised. However, more women were supervised than men. We can speculate that such gender finding can be attributed to the sport performed. Indeed, 35.8% of women performed dance/gymnastics or general fitness programs against only 4.6% of men. These sports are well known to include extensive stretching programs for flexibility improvements [68,69]. Obviously, coaches or physical trainers mostly supervised stretching exercises. In addition, national/international athletes were more supervised than regional or recreational individuals. This level dependency was not surprising since performing elite sport imposed more coaching professionalism with an exhaustive view of all training components. However, despite this positive information, only 50% of national/international athletes were supervised during stretching sessions. According to previous studies, supervision did not warrant appropriate practices. Authors have previously acknowledged that coaches could be hesitant to change their habits (for example while suppressing static stretching from pre-activity routines) [39,62]. Moreover, adequate supervision of stretching exercises (technique, volume, intensity) may be arduous with large groups of athletes [39].

Although 62.9% of the individuals were not supervised, a large proportion (76.2%) received instructions from coaches, physical trainers or health professionals. As for supervision, a practice level dependency was observed with more national/international athletes receiving instructions. This result was apparently encouraging for better and adequate stretching interventions. However, even with instructions, the predominant absence of supervision did not guarantee individuals would understand or follow the general recommendations or use appropriate exercises and correct techniques or positioning [70,71,72]. Supervision should be developed since it is well known to increase training efficiency [73,74].

Most individuals acknowledged the existence of different stretching modalities. The preferred and generally used stretching technique was static followed by dynamic stretching. In the present survey, general PNF techniques was intentionally divided into PNF, contract-relax (concentric contraction preceding a stretch) and hold-relax (isometric contraction preceding a stretch). This apparent dupery revealed that respondents did not know the term PNF whilst they seemed to practice the techniques. Previous studies demonstrated different efficiency between stretching techniques [14,15,16,17,18,19]. However, other studies failed to demonstrate any gold method to gain flexibility in sport or for rehabilitation [75,76]. Considering the general lack of supervision, we could recommend individuals to use the simplest stretching techniques or the technique they usually practice avoiding inappropriate intensity and positioning.

### 4.3. Study Limitations

Some limitations should be acknowledged. The data were collected using a self-reported survey. It could include potential bias related to subjective aspects, terminology understandings and generalization of the practices. Because this survey aimed to have an overview of a general stretching practices, the specific and very detailed stretching processes were not determined here. Detailed stretching exercises, duration, and intensity would have been of interest. Moreover, although the present survey was completed by 3546 individuals, more respondents would have increased the strength of our conclusions. Increasing the number of respondents would have permitted to discriminate practices between age, type of sport or physical activity. Further investigations should be conducted to determine the stretching practices and culture in depth in specific sports.

## 5. Conclusions

From the present survey, we concluded that stretching practices were partly in agreement with the literature, for example while considering the modality used for performance. In contrast and in disagreement with the recent literature, most individuals indicated they perform stretching for recovery reasons. Education, instructions, and supervision should be developed to favor appropriate stretching intensity, technique, and positioning. Indeed, from the present survey, supervision appeared poorly provided. Elite competitive individuals appeared more supervised and conducted slightly more adequate and evidence-based stretching sessions. Other gender differences were noticed that could be attributed to the practiced sport and subjective flexibility.

## Figures and Tables

**Table 1:** Participants’ characteristics.

	Gender ^$$$^		Practice level ^£££^		
Descriptor	Women	Men	Nat./Internat.	Regional	Recreative
What is your gender?					
Women ^b,c,d^	47.3% (1677)	-	41.3% (371)	34.4% (364)	59.2% (942)
Men ^b,c,d^	-	52.7% (1869)	58.7% (527)	65.6% (693)	40.8% (549)
What is your age?					
<20 years ^a,b,c,d^	32.8% (550)	24.9% (465)	37.3% (335)	30.4% (322)	22.5% (358)
20–29 years	51.2% (859)	48.1% (899)	50.3% (452)	47.5% (502)	50.5% (804)
30–39 years ^a,c^	6.4% (107)	9.8% (183)	6.8% (61)	9.5% (101)	8.0% (128)
40–49 years ^a,b,c,d^	5.1% (85)	10.0% (187)	4.0% (36)	7.6% (80)	9.8% (80)
50–59 years ^a,b,c,d^	3.9% (65)	5.3% (99)	1.4% (13)	3.9% (42)	6.8% (42)
>60 years ^a,b,c,d^	0.6% (11)	1.9% (36)	0.1% (1)	0.9% (10)	2.2% (10)
What is the main sport you are actually doing?					
Team Sports ^a,b,c,d^	18.3% (307)	40.8% (762)	43.5% (391)	55.0% (581)	6.1% (97)
Fitness ^a,b,c,d^	21.3% (357)	3.1% (58)	0.4% (4)	0.8% (8)	25.3% (403)
Strength/crossfit ^a,c,d^	8.6% (144)	11.3% (211)	1.8% (16)	2.2% (23)	19.9% (316)
Track & Field	9.2% (154)	8.7% (162)	9.8% (88)	9.6% (101)	8.0% (127)
Cycling/Trail/Triathlon ^a,b,c,d^	7.1% (120)	10.5% (196)	7.1% (64)	5.8% (61)	12.0% (191)
Racket Sport ^a,b,c,d^	5.3% (88)	11.0% (206)	6.7% (60)	15.1% (160)	4.7% (74)
Dance/Gymnastics ^a,b,d^	14.5% (243)	1.5% (29)	10.4% (93)	3.0% (32)	9.2% (147)
Martial Arts ^b,c,d^	4.8% (80)	5.2% (98)	8.7% (78)	2.6% (27)	4.6% (73)
Swimming	3.3% (56)	2.3% (44)	2.8% (25)	2.4% (25)	3.1% (50)
Mountain/water sports ^b,d^	2.6% (44)	3.0% (57)	4.5% (40)	0.9% (9)	3.3% (52)
Equestrian/Golf ^a^	3.5% (59)	0.6% (11)	2.2% (20)	2.1% (22)	1.8% (28)
Others ^b,d^	1.5% (25)	1.9% (35)	2.1% (19)	0.8% (8)	2.1% (33)
What is your practice level?					
National/international ^a^	22.2% (371)	28.2% (527)	-	-	-
Regional ^a^	21.7% (364)	37.1% (693)	-	-	-
Recreative ^a^	56.2% (942)	34.7% (649)	-	-	-
What is your training volume?					
<2 h/week ^a,b,c,d^	21.3% (357)	14.6% (273)	6.1% (55)	14.6% (154)	26.5% (421)
3–6 h/week ^a,c,d^	44.9% (754)	38.6% (722)	27.7% (249)	42.2% (446)	49.1% (781)
7–10 h/week ^a,b,c,d^	22.2% (373)	26.6% (497)	28.6% (257)	31.0% (328)	17.9% (285)
>10 h/week ^a,b,c,d^	11.5% (193)	20.2% (377)	37.5% (337)	12.2% (129)	6.5% (104)
What is your subjective flexibility?					
Very low ^a^	12.2% (205)	22.5% (421)	13.8% (124)	22.1% (234)	16.8% (268)
Low ^a,b,d^	36.9% (619)	45.1% (843)	39.5% (355)	46.5% (491)	38.7% (616)
High ^a,b,d^	41.1% (690)	28.9% (540)	37.6% (338)	27.4% (290)	37.8% (602)
Very high ^a^	9.7% (163)	3.5% (65)	9.0% (81)	4.0% (42)	6.6% (105)

Values are presented as percentages and number of respondents (*n*). Significant frequency distribution differences between men and women ($$$) or between practice levels (^£££^) for all questions (*p* < 0.001). Significant differences using Z-scores for a given item between (^a^) women and men, (^b^) nat./internat. and regional, (^c^) nat./internat. and recreative and (^d^) regional and recreative (*p* < 0.05). Nat./Internat.: national/international level.

**Table 2:** General stretching practices.

	Gender ^$$$^		Practice level ^£££^		
Descriptor	Women	Men	Nat./Internat.	Regional	Recreative
For what reason? *					
Wellness ^a,b,c,d^	56.2% (850)	43.4% (714)	14.3% (386)	14.1% (393)	18.7% (789)
Warm-up ^b,c,d^	50.3% (761)	49.6% (821)	17.2% (463)	17.7% (494)	14.8% (626)
Injury prevention ^a,c,d^	51.4% (778)	58.8% (973)	19.3% (521)	20.3% (567)	15.5% (654)
Gain flexibility ^b,c,d^	59.1% (894)	55.5% (919)	19.3% (520)	17.3% (484)	19.2% (810)
Recovery ^a,b,c^	81.0% (1226)	69.3% (1147)	24.1% (650)	24.4% (682)	24.6% (1040)
Health	19.1% (289)	20.3% (336)	5.8% (157)	6.1% (171)	7.1% (302)
When? *					
Before training ^b,c,d^	44.7% (676)	45.3% (750)	19.6% (440)	19.3% (434)	19.7% (550)
During training ^c,d^	17.4% (263)	19.4% (322)	8.6% (193)	8.6% (193)	7.3% (203)
After training/competition ^a,b,c^	76.1% (1152)	68.9% (1141)	28.6% (641)	30.3% (681)	34.7% (970)
After series of training/competition ^b,c,d^	30.9% (468)	32.9% (544)	18.8% (422)	17.3% (388)	11.9% (334)
Dedicated sessions ^b,c,d^	49.2% (745)	51.0% (845)	24.4% (547)	24.4% (549)	26.3% (736)
With what frequency?					
Every day ^b,c,d^	10.3% (156)	9.0% (150)	13.6% (115)	6.4% (61)	9.4% (130)
During every training ^c^	28.6% (432)	24.5% (407)	21.8% (184)	26.3% (250)	29.4% (405)
1 to 5 times a week	43.4% (655)	43.5% (722)	43.0% (363)	42.9% (408)	44.1% (607)
1 to 2 times per month ^c,d^	13.5% (204)	17.4% (289)	17.2% (145)	18.3% (174)	12.6% (174)
1 to 6 times per year ^d^	4.2% (63)	5.6% (93)	4.3% (36)	6.1% (58)	4.4% (61)
What is the average duration of stretching exercises (total)?					
<15 min ^c,d^	41.2% (623)	40.6% (712)	47.8% (403)	54.7% (520)	29.9% (412)
Between 15 and 30 min	27.3% (412)	27.4% (480)	24.9% (210)	28.1% (267)	30.1% (415)
Between 30 and 60 min ^b,c,d^	29.5% (446)	30.7% (539)	26.1% (220)	16.1% (153)	37.6% (518)
>60 min	2.0% (30)	1.3% (23)	1.2% (10)	1.2% (11)	2.3% (32)
What part of your body?					
Lower ^a^	19.6% (277)	29.8% (494)	25.8% (217)	18.9% (294)	30.1% (260)
Upper ^a^	0.8% (12)	1.8% (29)	1.5% (13)	1.2% (44)	1.1% (17)
Both ^a^	79.5% (1124)	68.4% (1132)	72.7% (612)	79.8% (646)	67.9% (1098)

Values are presented as percentages and number of respondents (*n*). Questions with potential multiple responses are shown (*). ^$$$^ significant frequency distribution differences between women and men except for stretching frequency and duration (*p* < 0.001). £££ significant frequency distribution differences between practice levels for all questions (*p* < 0.001). Significant differences using Z-scores for a given item between (^a^) women and men, (^b^) nat./internat. and regional, (^c^) nat./internat. and recreative and (^d^) regional and recreative (*p* < 0.05). Nat./Internat.: national/international level.

**Table 3:** Stretching and injury.

	Gender ^$$^		Practice level ^££^		
Descriptor	Women	Men	Nat./Internat.	Regional	Recreative
Did you get injured during the last 12 months?					
Yes ^a,c,d^	40.7% (683)	49.0% (915)	57.2% (514)	54.7% (579)	31.7% (505)
No ^a,c,d^	59.3% (994)	51.0% (954)	42.7% (384)	45.2% (478)	68.2% (1086)
Do you think stretching more could have avoided being injured? (in case of injuries)					
Yes ^a,b,c,d^	26.2% (178)	36.0% (329)	26.8% (138)	34.5% (198)	33.9% (171)
No ^b,c,d^	51.0% (346)	44.8% (409)	52.9% (272)	46.8% (269)	42.4% (214)
No opinion ^c,d^	22.8% (155)	19.2% (175)	20.2% (104)	18.6% (107)	23.6% (119)
Do you think stretching contribute to the absence of injury? (if no injury)					
Yes ^a^	45.6% (452)	41.9% (398)	47.6% (183)	42.7% (204)	42.8% (463)
No ^a^	12.4% (123)	18.0% (171)	14.5% (56)	17.6% (84)	14.2% (154)
May be ^a^	42.0% (416)	40.2% (382)	37.7% (145)	39.6% (189)	42.9% (464)

Values are presented as percentages and number of respondents (*n*). ^$$^ significant frequency distribution differences between men and women for all questions (*p* < 0.01). ^££^ significant frequency distribution differences between practice level except for the last question (*p* < 0.01). Significant differences using Z-scores for a given item between (^a^) women and men, (^b^) nat./internat. and regional, (^c^) nat./internat. and recreative and (^d^) regional and recreative (*p* < 0.05). Nat./Internat.: national/international level.

## Data Availability

The authors declare that the dataset is available on request.

## Supplementary Materials

The following is available online at https://www.mdpi.com/article/10.3390/ijerph18083928/s1, Table S1: Questionnaire used during the present study. Table S2: Effect sizes from Chi-square analyses (Cramer’s V).
