# Supplementary material for: A Survey on Stretching Practices in Women and Men from Various Sports or Physical Activity Programs
Source: Int J Environ Res Public Health. 2021 Apr 8;18(8):3928. doi: 10.3390/ijerph18083928 (PMC8068839; doi:10.3390/ijerph18083928)
Supplement: Supplementary file 1 [file ijerph-18-03928-s001.pdf]

Table S1. Questionnaire used during the present study.

Questions	Responses	Other Information
Q1—What is your gender?	Women, Men	
Q2—What is your age?	<20 years, 20–29 years, 30–39 years, 40–49 years, 50–59 years, >60 years	
Q3—What is the main sport you are actually doing?	Team Sports, Fitness, Strength/crossfit, Track & Field, Cycling/Trail/Triathlon, Racket Sport, Dance/Gymnastics, Martial Arts, Swimming, Mountain/water sports, Equestrian/Golf, Others	
Q4—What is your practice level?	National/international, Regional, Recreative	
Q5—What is your training volume?	<2 h/week, 3–6 h/week, 7–10 h/week, >10 h/week	
Q6—What is your subjective flexibility?	Very low, Low, High, Very high	
Q7—Do you already feel the necessity to do stretching exercises?	Yes, No	
Q8—For what reason?	Back pain, Joint pain, Muscle pain, Muscle stiffness, Range of motion, Wellness, Others	If Q7 is yes<br>* multiple responses
Q9—When?	Before training, During training, After training/competition, After series of training/competition, Dedicated sessions	If Q7 is yes<br>* multiple responses
Q10—Did you conduct stretching exercises during the last two years?	Yes, No	
Q11—For what reason?	No time, No motivation, No information, no supervision, poor efficiency, others	If Q10 is no<br>* multiple responses
Q12—For what reason?	Wellness, Warm-up, Injury prevention, Gain flexibility, Recovery, Health, Others	If Q10 is yes<br>* multiple responses
Q13—When?	Before training, During training, After training/competition, After series of training/competition, Dedicated sessions	If Q10 is yes<br>* multiple responses
Q14—With what frequency?	Every day, During every training, 1 to 5 times a week, 1 to 2 times per month, 1 to 6 times per year	If Q10 is yes
Q15—What is the average duration of stretching exercises (total)?	<15 min, Between 15 and 30 min, Between 30 and 60 min, > 60 min	If Q10 is yes
Q16—What part of your body?	Lower, Upper, Both	If Q10 is yes
Q17—Did you receive any information about stretching during your education?	Yes, No	
Q18—Did you look at some documentation to help you understand and perform stretching?	Yes, No	
Q19—What is a type of documentation you obtained?	Education, Coach/health professional, Conversation, Internet, Video, Books, Others	If Q18 is yes<br>* multiple responses
Q20—Are stretching exercises supervised?	Yes, No	
Q21 - Who is the supervisor?	Other athletes, Coach, Health professional, Others	If Q20 is yes<br>* multiple responses
Q22—Do you have instruction to perform stretching?	Yes, No	
Q23—Who are the people giving instruction?	Other athletes, education, Coach, Health professional, Others	If Q22 is yes<br>* multiple responses
Q24—Do you know different stretching modalities?	Yes, No	
Q25—What stretching modality do you generally used?	Active, Passive, Static, Dynamic, Ballistic, Oscillations, PNF, Contract-relax, Hold-relax, Others	If Q24 is yes<br>* multiple responses
Q26—What stretching modality do you use for performance?	Active, Passive, Static, Dynamic, Ballistic, Oscillations, PNF, Contract-relax, Hold-relax, Others	If Q24 is yes<br>* multiple responses
Q27—What stretching modality do you use for recovery?	Active, Passive, Static, Dynamic, Ballistic, Oscillations, PNF, Contract-relax, Hold-relax, Others	If Q24 is yes<br>* multiple responses
Q28—What stretching modality do you use for wellness?	Active, Passive, Static, Dynamic, Ballistic, Oscillations, PNF, Contract-relax, Hold-relax, Others	If Q24 is yes<br>* multiple responses

Q29—What stretching modality do you use for flexibility?	Active, Passive, Static, Dynamic, Ballistic, Oscillations, PNF, Contract-relax, Hold-relax, Others	If Q24 is yes<br>* multiple responses
Q30—Did you get injured during the last 12 months?	Yes, No	
Q31—Do you think stretching more could have avoided being injured?	Yes, No, No opinion	If Q30 is yes
Q32—Do you think stretching contribute to the absence of injury?	Yes, No, Maybe	If Q30 is no

Table S2. Effect sizes from Chi-square analyses (Cramer's V).

Questions	Gender	Practice Level
Q1—What is your gender?	-	0.221
Q2—What is your age?	0.142	0.139
Q3—What is the main sport you are actually doing?	0.440	0.467
Q4—What is your practice level?	0.221	-
Q5—What is your training volume?	0.149	0.294
Q6—What is your subjective flexibility?	0.212	0.101
Q7—Do you already feel the necessity to do stretching exercises?	0.056	0.033
Q8—For what reason?	0.103	0.121
Q9—When?	0.094	0.067
Q10—Did you conduct stretching exercises during the last two years?	0.027	0.097
Q11—For what reason?	0.019	0.039
Q12—For what reason?	0.075	0.071
Q13—When?	0.102	0.068
Q14—With what frequency?	0.073	0.092
Q15—What is the average duration of stretching exercises (total)?	0.05	0.105
Q16—What part of your body?	0.144	0.085
Q17—Did you receive any information about stretching during your education?	0.136	0.049
Q18—Did you look at some documentation to help you understand and perform stretching?	0.116	0.008
Q19—What is a type of documentation you obtained?	0.197	0.184
Q20—Are stretching exercises supervised?	0.128	0.172
Q21—Who is the supervisor?	0.223	0.221
Q22—Do you have instruction to perform stretching?	0.008	0.062
Q23—Who are the people giving instruction?	0.206	0.198
Q24—Do you know different stretching modalities?	0.235	0.267
Q25—What stretching modality do you generally used?	0.066	0.087
Q26—What stretching modality do you use for performance?	0.122	0.143
Q27—What stretching modality do you use for recovery?	0.085	0.076
Q28—What stretching modality do you use for wellness?	0.089	0.093
Q29—What stretching modality do you use for flexibility?	0.144	0.139
Q30—Did you get injured during the last 12 months?	0.083	0.242
Q31—Do you think stretching more could have avoided being injured?	0.105	0.069
Q32—Do you think stretching contribute to the absence of injury?	0.078	0.041
